# Early maladaptive schemas and their relation to personality disorders: A correlational examination in a clinical population

**DOI:** 10.1002/cpp.2467

**Published:** 2020-06-17

**Authors:** Hannah Kunst, Jill Lobbestael, Ingrid Candel, Tim Batink

**Affiliations:** ^1^ Department of Clinical Psychological Science, Faculty of Psychology and Neuroscience Maastricht University The Netherlands; ^2^ U‐Center Heerlen The Netherlands; ^3^ Present address: The University of Sydney NSW Australia; ^4^ Present address: Open Universiteit Epen AH The Netherlands

**Keywords:** borderline, dimension, early maladaptive schemas, hybrid model, personality disorder

## Abstract

Personality disorder (PD) pathology has been linked to early maladaptive schemas (EMSs). Because of a large heterogeneity in study populations, sample size, statistical analyses and conceptualizations in the literature, the exact relationships between PDs and EMSs are still unclear. The current study examined the relationship between borderline, dependent, avoidant and obsessive–compulsive PDs, represented dimensionally as number of traits, and 15 different EMSs as measured by the Young Schema Questionnaire (YSQ). A total of *N* = 130 inpatients took part in the study (*M* ^age^ = 43.6, gender = 51.5% female). Stepwise regressions indicated that borderline, dependent, avoidant and obsessive–compulsive PD traits were partly characterized by specific EMSs and EMSs grouped as domains (i.e., other‐directedness domain for dependent PD and overvigilance for obsessive–compulsive PD) and that relations with a variety of domains and EMSs were overlapping for the PD dimensions (i.e., disconnection and rejection for both borderline and avoidant PDs). This suggests that PDs are reflected by a hybrid model of EMSs, with some EMSs and domains that relate to a broader vulnerability factor for PDs, and other domains that differentially relate to the independent PDs. Findings are informative for clinicians, as various EMSs per PD may be targeted in therapy.

**Key Practitioner Messages**

- Personality disorders (PDs) are characterized by a hybrid model with some early maladaptive schemas as broad vulnerability factors, and others being PD‐specific.
- Early maladaptive schemas can be effectively related across various PD dimensions.
- Treatment targeting various schemas per PD is suitable.

## 1 INTRODUCTION

Personality disorders (PDs) refer to pervasive, persistent and pathological patterns of perception and behaviour. These patterns deviate from expectations of the individuals' culture and manifest themselves in cognition, affectivity, interpersonal functioning and/or impulse control. Currently, 10 PDs are identified in the Diagnostic and Statistical Manual of Mental Disorders (DSM‐5; American Psychiatric Association [APA], 2013) and indicated by at least four and up to nine features (i.e., ‘frantic efforts to avoid real or imagined abandonment’ or ‘chronic feelings of emptiness’ for borderline PD, p. 663). Various clinical interventions, including Interpersonal Reconstructive Therapy (Critchfield, Benjamin, & Levenick, 2015), Metacognitive Interpersonal Therapy (Dimaggio et al., 2017) and Schema‐Focused Therapy (Nordahl, Holthe, & Haugum, 2005), assume that schemas are trait concepts that are at the core of PDs. Schemas refer to cognitive structures and dysfunctional belief systems (Kellogg & Young, 2006). Within Schema‐Focused Therapy, PD pathology is linked to early maladaptive schemas (EMSs; Young, 1999). Following Young's conceptualization (Young, 1999), EMSs are strong, stable beliefs regarding self, others and the world. They develop early in childhood, and relate to childhood adversity (Lumley & Harkness, 2007) and parental rearing styles (Muris, 2006; Thimm, 2010), but also to temperament, character traits (Halvorsen et al., 2009) and attachment styles (Simard, Moss, & Pascuzzo, 2011). The different EMSs become activated by certain events or situations. This leads to experiences of intense affect and the use of (maladaptive) coping strategies (Mairet, Boag, & Warburton, 2014), which may directly or indirectly cause psychological distress and pathology. EMSs moreover filter the individual's experiences and guide interactions with others and the environment, in turn further confirming the beliefs linked to the EMS. This confirmation strengthens EMSs over time and leads to further distress and negative thoughts resistant to change (Jovev & Jackson, 2004). Up until now, 18 different EMSs have been defined, which can be divided into five domains: disconnection and rejection, impaired autonomy, other‐directedness, overvigilance and impaired limits (see Supporting Information for descriptions of the individual EMSs).

Correlational research has identified many relations between EMSs and the different PDs (see Lobbestael & Arntz, 2012, for a tabular overview). A literature search (search terms on PsycINFO and PubMed between 1982 and 2019 included ‘personality disorder’, ‘relation’, ‘early maladaptive schema’ and ‘schema domain’) was conducted, which identified 15 studies that used clinical diagnostic measures for PD identification. The majority of this PD literature has focused on borderline PD. Out of the 15 studies, 14 included borderline PD in their analysis. Furthermore, eight studies investigated the link between EMSs and avoidant PD, seven focused on antisocial PD, and five on paranoid, schizoid, schizotypal, dependent, obsessive–compulsive, narcissistic and histrionic PDs. More specifically, for paranoid PD, most studies found a relation with mistrust/abuse and social isolation EMSs (Carr & Francis, 2010; Gilbert & Daffern, 2013; Nordahl et al., 2005; Parveen, Shehzadi, Riaz, & Yasmin, 2017; Reeves & Taylor, 2007). Schizoid and schizotypal PDs were most often reported to relate with social isolation (Gilbert & Daffern, 2013; Reeves & Taylor, 2007), and histrionic PD related frequently to entitlement (Carr & Francis, 2010; Nordahl et al., 2005). Antisocial PD related to mistrust/abuse, vulnerability to harm and emotional inhibition EMSs (Ball & Cecero, 2001; Özdel et al., 2015). Borderline PD was most often represented in the literature, with studies reporting relations across all 18 EMSs (Bach & Farrell, 2018; Flink et al., 2018; Hulbert, Jennings, Jackson, & Chanen, 2011; Jovev & Jackson, 2004; Lawrence, Allen, & Chanen, 2011; Meyer, Leung, Feary, & Mann, 2001; Nilsson, Jørgensen, Straarup, & Licht, 2010). Narcissistic PD was found to relate to entitlement EMS across studies (Carr & Francis, 2010; Reeves & Taylor, 2007; Zeigler‐Hill, Green, Arnau, Sisemore, & Myers, 2011). Avoidant PD was represented mostly by relations with subjugation and emotional inhibition (Carr & Francis, 2010; Gilbert & Daffern, 2013; Nordahl et al., 2005). Dependent PD related most to abandonment/instability (Carr & Francis, 2010; Nordahl et al., 2005; Reeves & Taylor, 2007) and obsessive–compulsive PD to unrelenting standards (Carr & Francis, 2010; Jovev & Jackson, 2004; Reeves & Taylor, 2007). Findings on the relations between specific EMSs and PDs vary largely between studies. For example, Thimm (2010) and Shorey, Anderson, and Stuart (2014) reported relations of borderline PD with EMSs from all five domains, whereas Carr and Francis (2010) did not find significant relations between borderline PD and any of the EMSs.

Inconsistent findings might be the result of several methodological differences within the literature. First of all, there is a large variety of study populations within the EMS–PD literature, including clinical, healthy, incarcerated and youth populations. Four out of the 15 studies used a mixed clinical sample with clinical diagnoses of various PDs, albeit all from an outpatient setting. Sample sizes vary widely too, that is, ranging from *N* = 13 (Jovev & Jackson, 2004) to *N* = 804 (Reeves & Taylor, 2007). Often, studies only looked at the EMS domains instead of separate EMSs (e.g., Corral & Calvete, 2014; Loper, 2003; Shorey et al., 2014; Specht, Chapman, & Cellucci, 2009; Thimm, 2010). Finally, there is a large variety in power and statistical analyses. Many studies for example only reported *t‐*tests comparing mean EMS scores of certain PD groups versus other clinical or PD groups (Bach & Farrell, 2018; Flink et al., 2018; Hulbert et al., 2011; Jovev & Jackson, 2004) or non‐patient controls (Lawrence et al., 2011; Nilsson et al., 2010; Özdel et al., 2015). Likewise, some studies conducted cluster analysis (Petrocelli, Glasner, Calhoun, & Campbell, 2001) and path analysis (Corral & Calvete, 2014) without reporting correlations or grouped all EMSs into one omnibus outcome variable (Batool, Shehzadi, Riaz, & Riaz, 2017; Khodarahimi, 2017). A final noteworthy distinction in study approaches on the EMS–PD relationship concerns the conceptualization of PDs. Specifically, in some of the studies, PDs are operationalized by a dimensional severity indication based on the number of PD traits (Carr & Francis, 2010; Dimaggio et al., 2013; Mącik, 2018; Meyer et al., 2001; Parveen et al., 2017; Reeves & Taylor, 2007) or symptom severity (Gilbert & Daffern, 2013), whereas other studies take a categorical PD approach. These different approaches limit the generalizability of findings on the EMS–PD relationship. Gaining more insight is however of clinical importance, as identification of underlying EMSs is a critical component in the treatment of PDs (Jovev & Jackson, 2004).

### 1.1 The current study

Because of the large heterogeneity in study populations, sample size, statistical analyses and conceptualizations of EMSs (domains vs. separate EMSs) and PDs (categorical vs. dimensional), the exact relationships between PDs and EMSs are currently still unclear. It has been empirically established that both PDs and EMSs are better represented as dimensional instead of categorical constructs. Limitations regarding the existing diagnostic PD categories, including heterogeneity of patients with the same diagnosis, diagnostic co‐occurrence, unstable diagnostic boundaries with normal psychological functioning and inadequate coverage, including diagnosis of not otherwise specified (Widiger & Trull, 2007), aid arguments towards a dimensional approach. Accordingly, it was stated in the DSM‐5 that ‘the benefits of a more dimensional approach to PDs have been identified’ (p. 43) and as such an alternative hybrid model next to the categorical diagnostic system, including a more dimensional profile of trait expression for a trait‐specified approach, was provided. Arntz et al. (2009) examined the latent structure of avoidant, dependent, obsessive–compulsive, depressive, paranoid and borderline PDs using taxometric analyses and found greater evidence for a latent dimensional structure than for taxonic simulations. Dimaggio et al. (2013) furthermore found that a higher number of PD traits related to increased symptom severity and interpersonal problems, further supporting the added value of examining PDs by trait expression. A focus on a dimensional over a categorical approach regarding PDs and EMSs therefore seems warranted. The current study sets out to examine the relationship between PDs, represented dimensionally as number of traits, and 15 different EMSs. Overcoming previous limitations, the current study includes a large, clinical inpatient population with PD traits and diagnoses assessed by clinicians. All separate EMSs instead of domains will be examined to establish more specific EMS–PD relations, and statistical analyses will not be limited to uncorrected correlations. Due to current sample characteristics (i.e., low prevalence and nonnormal distribution of several PDs, see Section 2), the present study will focus on borderline, avoidant, dependent and obsessive–compulsive PDs.

Based on previous findings, it is hypothesized that PDs relate to various EMSs. Specifically, it is hypothesized that (1) borderline PD traits mostly relate to the abandonment/instability, mistrust/abuse, emotional deprivation, defectiveness and social isolation EMSs, (2) avoidant PD traits mostly relate to subjugation and emotional inhibition EMSs, (3) dependent PD traits mostly relate to abandonment/instability EMS and (4) obsessive–compulsive PD traits mostly relate to unrelenting standards EMS. Furthermore, as there are established gender differences (Paris, 2004) and high co‐morbidity in PDs (McGlashan et al., 2000), as well as influences of age, education and gender on the level of reported EMSs (Bora & Baysan Arabaci, 2009; Pellerone, Iacolino, Mannino, Formica, & Zabbara, 2017), the current study will statistically control for age, education, gender and PD traits.

## 2 METHOD

### 2.1 Participants

A total of *N* = 130 inpatients from a psychological health care centre in the south of the Netherlands took part in the study. The centre specializes in the treatment of psychiatric disorders such as depression, anxiety, addiction and PDs. Recruitment was conducted between 03‐12‐2015 and 06‐06‐2016. Of the sample, 51.5% was female, and the average age was 43.6 years (*SD* = 13.5, range = 19–86). Regarding education level, 1.5% (*n* = 2) attended primary school, 24.6% (*n* = 32) high school/low vocational studies, 23.8% (*n* = 31) secondary education and 38.5% (*n* = 50) completed a higher education. Regarding clinical diagnoses (based on the Mini International Neuropsychiatric Interview [MINI Plus 5.0.0]; Sheehan, Lecrubier, Sheehan, Amorim, & Janavs, 1998), 2.3% met the diagnostic criteria of an eating disorder, 67.7% of a mood disorder, 10.8% of a psychotic disorder, 49.2% of a substance‐related and addictive disorder, 63.8% of an anxiety disorder and 10.8% of a somatoform disorder. In total, 3.8% (*n* = 5) received no clinical diagnosis. Note that 97 participants had two or more clinical disorders.

### 2.2 Measures

#### 2.2.1 Mini International Neuropsychiatric Interview

The Mini International Neuropsychiatric Interview (MINI; Van Vliet & De Beurs, 2007) is a structured diagnostic interview, assessing 17 clinical diagnoses based on the DSM‐IV‐TR. Questions are answered with ‘yes’ or ‘no’. Disorders are ruled out when one or two questions are answered with no. The interrater reliability is generally acceptable, with *κ* = .36–.81 (Lecrubier et al., 1997).

#### 2.2.2 Structured Interview for DSM‐IV Personality

The Structured Interview for DSM‐IV Personality (SIDP‐IV; Pfohl, Blum, & Zimmerman, 1997) is a semistructured interview, measuring PD traits. It is used for diagnosing 10 distinct PDs based on the DSM‐IV‐TR. The SIDP‐IV consists of 96 questions, clustered in 10 categories, and five additional questions for observation purposes. All questions are based on a DSM‐IV‐TR criterion, and answers are scored on a scale ranging from 0 (*not present*) to 3 (*strongly present*). In the current study, dimensional scores corresponding to PD symptom severity were calculated by summing the scores (0, 1, 2, 3) in order for the item scores to be as differentiated as possible. Internal consistency of the SIDP‐IV has been established and found to be modest (with Cronbach's alpha of the subscales ranging from *α* = .66 to .82; Blais & Norman, 1997). The interrater reliability of de Dutch version of SIDP‐IV is fairly good, with *κ* = .66–1(Damen, de Jong, & van der Kroft, 2004).

#### 2.2.3 Young Schema Questionnaire

The Young Schema Questionnaire (Young & Brown, 1994) is a self‐report measure assessing 15 different EMSs. The YSQ consists of 205 items that are clustered in five domains (see Table 1). Answers are scored on a 6‐point Likert scale, ranging from 1 (*totally disagree*) to 6 (*totally agree*). The average score of each schema is calculated by summing the outcomes of all related items and then dividing this by the total number of questions. The Dutch version of the YSQ has high internal reliability with Cronbach's alpha between *α* = .74 and .92 (Rijkeboer & van den Bergh, 2006). Test–retest reliability for the different subscales ranged from *r* = .63 to .97 (Rijkeboer, van den Bergh, & van den Bout, 2005). Cronbach's alpha in the current study ranged from *α* = .52 for schizotypal PD to *α* = .83 for avoidant PD.

**TABLE 1:** YSQ descriptives

Domain	EMSs	*M*	*SD*	Range	Min.	Max.
Disconnection/rejection	Abandonment/instability	2.77	.91	4.33	1.06	5.39
	Mistrust/abuse	2.43	.85	3.65	1.00	4.65
	Emotional deprivation	2.53	1.04	4.89	1.00	5.89
	Defectiveness/Shame	2.37	.93	4.13	1.00	5.13
	Social isolation/alienation	2.98	1.07	4.40	1.00	5.40
Impaired autonomy/performance	Dependence/incompetence	2.84	.92	4.13	1.00	4.13
	Vulnerability to harm/illness	2.68	.94	4.57	1.00	5.57
	Enmeshment	2.33	.97	4.00	1.00	5.00
	Failure	2.92	1.09	4.44	1.00	5.44
Impaired limits	Entitlement	2.54	.73	4.18	1.00	5.18
	Insufficient self‐control	2.93	.89	4.54	1.13	5.67
Other‐directedness	Subjugation	2.78	.97	4.60	1.00	5.60
	Self‐sacrifice	3.42	.87	4.52	1.24	5.76
Overvigilance/inhibition	Emotional inhibition	2.68	.94	5.00	1.00	6.00
	Unrelenting standards	3.27	1.04	4.88	1.00	5.88

*Note*: *N* = 130.

Abbreviations: EMS, early maladaptive schema; YSQ, Young Schema Questionnaire.

### 2.3 Procedure

Data were acquired from inpatients admitted to U‐Center, a health care centre in the Netherlands. Completion of the YSQ and MINI is part of the standard intake procedure of each patient before hospitalization. The SIDP‐IV is conducted only when the presence of personality problems is suspected. The diagnostic evaluations were conducted by trained and experienced psychologists working at the centre. These data are stored in an electronic patient information file. Participants are asked for general consent to access their files for research purposes. All hospitalized patients who consented are included in the current study. Demographic variables, including participant number, gender, age and education level, were readily available and retrieved from the electronic patient information file.

### 2.4 Statistical analysis

Pearson correlations between the EMSs and PD traits, as well as age, gender and education level, were computed. Next, multiple (backward) stepwise regression analyses, with PD traits as predictors and EMSs as dependent variables, were conducted in order to examine the relations in more detail. As PD symptoms are highly interrelated (Carr & Francis, 2010), all four PDs were simultaneously included in the regressions as predictors. In total, 15 stepwise multiple regressions on the PD traits predictors were performed to examine the contribution of each of the EMSs for the different PDs, further taking age, education level and gender into consideration by including them as predictors. All analyses were conducted with SPSS 23.0 for windows (IBM Corp, 2013), with an alpha level set at 0.05. With variance inflation factors ranging from 1.044 to 1.455, collinearity was not deemed problematic (Field, 2013; O'Brien, 2007).

## 3 RESULTS

Of all participants, 53.8% (*n* = 70) was not diagnosed with a full‐blown PD. The remaining 46.2% (*n* = 60) was diagnosed with one or more PD, namely, avoidant (15.4%, *n* = 20), dependent (3.1%, *n* = 4), obsessive–compulsive (13.8%, *n* = 18), narcissistic (1.5%, *n* = 2), borderline (7.7%, *n* = 10), histrionic (0.8%, *n* = 1), antisocial (0.8%, *n* = 1), paranoid (1.5%, *n* = 2) or schizoid PD (0.8%, *n* = 1). None of the participants adhered to the criteria of schizotypal PD. Descriptive values of the YSQ are displayed in Table 1. The average score of all EMSs was *M* = 2.76, *SD* = 0.95 with a range of 2.33–3.42. At face validity, this seems comparable with other study outcomes using clinical populations (e.g., Flink et al., 2018, with range = 2.2–4.2; Gilbert & Daffern, 2013, with range = 2.08 – 3.46; Lee, Taylor, & Dunn, 1999, with range = 1.74 – 3.26). Descriptive statistics of PD traits are displayed in Table 2. Distributions of the EMSs and PD traits were examined for normality. Skewness and kurtosis for the YSQ items were under 1.96 and 7, respectively, suggesting that the distributions of the EMSs were in the acceptable range (Field, 2013). Skewness and kurtosis for the PD traits were more problematic (see Table 2). Paranoid, schizoid and schizotypal PD traits, as well as narcissistic, antisocial and histrionic PD traits, had non‐normal distributions and were very limited in number (*n* under 2) and as such excluded from further analysis. Borderline, avoidant, dependent and obsessive–compulsive PD traits remained included for further analyses.

**TABLE 2:** *M* (*SD*) and distribution of number of PD traits

PD traits	*M* (*SD*)	Skewness	Kurtosis
Paranoid	0.39 (0.89)	3.37	14.63
Schizoid	0.21 (0.61)	3.90	17.68
Schizotypal	0.17 (0.56)	4.10	19.87
Histrionic	0.35 (0.91)	3.31	13.55
Antisocial	1.62 (0.55)	4.22	20.86
Borderline	1.43 (1.73)	1.34	1.40
Narcissistic	0.48 (0.97)	2.70	7.95
Avoidant	1.33 (1.81)	1.34	.86
Dependent	0.99 (1.40)	1.53	1.65
Obsessive–compulsive	1.80 (1.49)	0.49	−.55

*Note*: *N* = 130.

Abbreviation: PD, personality disorder.

Clinical disorder severity (i.e., the summed score of MINI diagnoses) related to a higher number of PD traits, *t* = .36, *p* < .001. Age negatively and significantly correlated to clinical severity, *t* = −.31, *p* < .001, indicating that a higher age relates to lower clinical severity. This is in accordance with findings in the literature (Segal, Hook, & Coolidge, 2001).

Correlations of PD traits and EMSs are presented in Table 3. Avoidant and dependent PD traits were significantly and positively related to most of the 15 EMSs, with the exception of entitlement and vulnerability to harm and illness for both PDs, and emotional deprivation for dependent PD traits. Borderline PD traits related positively and significantly to all 15 EMSs. Obsessive–compulsive PD traits positively related to most EMSs (*p*s < .05), except for abandonment, emotional deprivation and mistrust/abuse. Correlations with PD severity, as quantified by total number of PD traits present, and EMSs are also presented in Table 3. All correlations were significant at the *p* < .001 level, with *r* ranging from .33 for vulnerability to harm/illness to .55 for subjugation.

**TABLE 3:** Zero‐order correlations PD traits and EMSs

	Borderline	Avoidant	Dependent	Obsessive–compulsive	Symptom severity
Dependence/incompetence	.37^**^	.25^**^	.31^**^	.23^**^	.42^**^
Failure	.35^**^	.48^**^	.41^**^	.20^*^	.45^**^
Social isolation/alienation	.41^**^	.46^**^	.21^*^	.28^**^	.51^**^
Defectiveness/shame	.43^**^	.43^**^	.25^**^	.21^*^	.50^**^
Abandonment/instability	.50^**^	.40^**^	.41^**^	.14	.50^**^
Emotional deprivation	.25^**^	.34^**^	.13	.16	.36^**^
Mistrust/abuse	.37^**^	.31^**^	.18^*^	.13	.42^**^
Insufficient self‐control/self‐discipline	.46^**^	.19^**^	.34^**^	.23^*^	.47^**^
Entitlement	.37^**^	.14	.13	.33^**^	.42^**^
Unrelenting standards	.23^**^	.18^*^	.24^**^	.53^**^	.38^**^
Emotional inhibition	.38^**^	.35^**^	.25^**^	.27^**^	.48^**^
Self‐sacrifice	.21^*^	.24^**^	.36^**^	.23^*^	.34^**^
Subjugation	.48^**^	.40^**^	.50^**^	.21^*^	.55^**^
Enmeshment	.32^**^	.34^**^	.38^**^	.20^*^	.40^**^
Vulnerability to harm and illness	.31^**^	.15	.17	.17^*^	.33^**^

*Note*: *N* = 130.

Abbreviations: EMS, early maladaptive schema; PD, personality disorder.

^*^ *p* < .05 (two‐tailed).

^**^ *p* < .001 (two‐tailed).

Table 4 shows the outcomes of the stepwise regression analyses examining the relationship between EMSs and each of the PD traits after controlling for age, education level, gender and other PD traits. Having conducted multiple testing, we maintained a conservative manner of data interpretation and will focus on the relations significant at the *p* < .001 level. Borderline PD traits were significantly and positively related to most EMSs. No significant relations were found for emotional deprivation, enmeshment, failure, self‐sacrifice and unrelenting standards. The avoidant PD dimension was positively associated with several EMSs from the disconnection and rejection domain, namely, emotional deprivation, defectiveness/shame and social isolation/alienation. Dependent PD traits were positively associated with both EMSs from the other‐directedness domain, subjugation and self‐sacrifice. Finally, obsessive–compulsive PD traits were positively related with the unrelenting standard EMS, as well as with entitlement.

**TABLE 4:** Standardized beta coefficients and significance level of multiple regression on four PD traits and 15 EMSs (*N* = 130)

	EMSs	BPD	AVPD	DPD	OCPD	Age	Gender	Education	*R* ^2^
Disconnection/rejection	A/I	.36^**^	.21^*^	.18	.03	.03	−.09	−.03	.35
	M/A	.31^**^	.21^*^	−.07	.04	.06	−.09	.02	.19
	E	.14	.38^**^	−.11	.10	−.01	−.03	−.05	.14
	D/S	.33^**^	.35^**^	−.05	.11	−.10	−.09	−.02	.31
	S/A	.27^*^	.34^**^	−.14	.17^*^	−.00	−.01	.02	.33
Impaired Autonomy/performance	D/I	.35^*^	.09	.15	.18^*^	−.04	−.14	−.08	.18
	V	.40^**^	.06	.10	.15	.16	−.19^*^	.14	.16
	Enm	.09	.20^*^	.22^*^	.09	−.21^*^	.02	.06	.23
	F	.13	.31^*^	.18	.09	−.24^*^	.02	.07	.31
Impaired limits	En	.32^**^	.06	−.10	.30^**^	−.04	−.12	.07	.23
	I/S	.42^**^	−.04	.21^*^	.13	−.12	−.19^*^	−.08	.28
Other‐directedness	Su	.33^**^	.17	.31^**^	.08	−.09	−.13	.03	.39
	S	.06	.07	.38^**^	.14	.05	−.03	−.13	.14
Overvigilance/inhibition	Em	.30^**^	.22^*^	.03	.21^*^	−.12	−.14	−.01	.27
	U	.12	−.05	.06	.52^**^	−.18^*^	−.01	−.02	.32

Abbreviations: A/I, abandonment/instability; AVPD, avoidant PD; BPD, borderline PD; D/I, dependence/incompetence; D/S, defectiveness/shame; DPD, dependent PD; E, emotional deprivation; Em, emotional inhibition; EMSs, early maladaptive schemas; En, entitlement; Enm, enmeshment; F, failure; I/S, insufficient self‐control/self‐discipline; M/A, mistrust/abuse; OCPD, obsessive–compulsive PD; PD, personality disorder; S, self‐sacrifice; S/A, social isolation/alienation; Su, subjugation; U, unrelenting standards; V, vulnerability to harm/illness.

^*^ *p* < .05 (two‐tailed),

^**^ *p* < .001 (two‐tailed).

Age was found to significantly and negatively relate to enmeshment, failure and unrelenting standards EMSs (*p*s < .05), with an older age relating to lower trait sores on these EMSs. Gender was also found to negatively and significantly influence the vulnerability to harm and insufficient self‐control/self‐discipline EMSs (*p'*s < .05), implying that males scored higher than females on these EMSs. Education did not relate to any of the EMSs.

## 4 DISCUSSION

The current study examined the relationship between PD traits and EMSs in a clinical population. Due to the limited range of traits present for most of the PDs in our sample, only four PDs were addressed in this study: borderline, avoidant, dependent and obsessive–compulsive PDs. The results showed that the total number of PD traits positively related to all 15 EMSs, and avoidant, dependent, borderline and obsessive–compulsive PD traits positively related to almost all EMSs, supporting the relevance of global severity (Hopwood et al., 2011) and trait‐based examination of PDs (Bach, Sellbom, Skjernov, & Simonsen, 2018).

Findings on the borderline PD dimension revealed significant and positive correlations with all of the 15 EMSs. This finding is in line with some studies (Meyer et al., 2001; Parveen et al., 2017), whereas others found significant relations with fewer EMSs (Gilbert & Daffern, 2013; Nordahl et al., 2005). Looking at unique relationships (i.e., after controlling for other PDs in hierarchical regression) between borderline PD and EMSs, Reeves and Taylor (2007) found abandonment, social isolation and enmeshment EMSs to significantly predict borderline PD. Mącik (2018) found relations with self‐sacrifice, insufficient self‐control and punitiveness, whereas Carr and Francis (2010) found no relation at all. Our study evidenced unique relations with abandonment/instability, mistrust/abuse, defectiveness/shame, vulnerability to harm, entitlement, insufficient self‐control, subjugation and emotional inhibition EMSs. Discrepancies in findings could be due to the fact that Reeves and Taylor (2007), Mącik (2018) and Carr and Francis (2010) relied on a non‐clinical student sample. The presence of more or more strongly pronounced EMSs in our clinical sample is therefore not unexpected. The positive relationship with the disconnection/rejection domain suits the first diagnostic borderline PD criterion of ‘frantic efforts to avoid real or imagined abandonment’ (APA, 2013). The second criterion, ‘a pattern of unstable and intense interpersonal relationships characterized by alternating between extremes of idealization and devaluation’, can explain the link to entitlement (i.e., devaluating others) and subjugation (i.e., avoiding abandonment).

Correlations of avoidant PD traits and EMSs were positive and significant for all EMSs except for entitlement and vulnerability to harm, alike previous studies (Gilbert & Daffern, 2013; Nordahl et al., 2005). Unique relations of avoidant PD were found for emotional deprivation, defectiveness/shame and social isolation/alienation. This is not in accordance with previous studies, where relations were found with emotional inhibition and entitlement (Carr & Francis, 2010; Reeves & Taylor, 2007). These studies relied on a non‐clinical student sample, as was the case with the discrepancies among findings for borderline PD. The unique relation with emotional inhibition was moreover significant in our study, albeit only at the *p* < .05 level. The positive relationship with emotional deprivation seems distinctive for the avoidant PD dimension, as this relation was not found for the other PDs in our sample. Emotional deprivation is characterized by absence of feelings of security and love, which may in turn be related to suppression of conflicts or low levels of emotional expression in families, often found in avoidant PD patients (Taylor, Laposa, & Alden, 2004).

Dependent PD traits significantly and positively correlated with all EMSs, except for emotional deprivation, entitlement and vulnerability to harm. This is partly in accordance with other studies, as similar but less significant relations with EMSs were reported (Gilbert & Daffern, 2013; Nordahl et al., 2005). When looking at unique relations, other studies found dependent PD to relate to the abandonment EMS only (Carr & Francis, 2010; Reeves & Taylor, 2007), whereas the current study evidenced unique relations with subjugation and self‐sacrifice. A relation with abandonment EMS was however found when looking at the zero‐order correlations. The relation with subjugation and self‐sacrifice is not surprising, as the other‐directedness domain largely corresponds to the central DSM‐5 criterion that describes dependent PD individuals to go to any length to get care and support from others and even volunteer to offer things that are unpleasant (APA, 2013).

Obsessive–compulsive PD traits correlated positively and significantly with al EMSs except for abandonment, emotional deprivation and mistrust/abuse. Other studies found similar but less (Nordahl et al., 2005) or no significant correlations at all (Gilbert & Daffern, 2013). Other studies moreover found unique relations with unrelenting standards EMS, as well as with enmeshment (Reeves & Taylor, 2007), defectiveness/shame and self‐sacrifice(Carr & Francis, 2010). Obsessive–compulsive PD traits did correlate with these EMSs in the current study, but unique relations were found only with entitlement and unrelenting standards EMSs. The latter reflects the need to strive to achieve high standards of behaviour or performance, which reflect traits often found in obsessive–compulsive PD behaviour (e.g., perfectionism, excessive devotion to work and rigidity; APA, 2013).

In conclusion, borderline and avoidant PDs showed the most unique relations with the EMSs while both dependent and obsessive–compulsive PD traits turned out to relate to far less EMSs. Borderline and avoidant PD traits further share a relationship with the disconnection/rejection domain (cf Loper, 2003, and Thimm, 2010). This finding may reflect the high level of co‐morbidity between borderline and avoidant PDs. Zanarini et al. (2004), for example, found that 59% of nonremitted borderline PD patients met criteria for avoidant PD and up to 16% in a remitted sample. The unique relations with EMSs of the disconnection/rejection domain did differ between avoidant and borderline PD traits, where only avoidant PD traits related to emotional deprivation. Similarities in cognitions and beliefs but different pathology expression could be explained by overlap in some, and differences in other risk factors for avoidant and borderline PD. Where both disorders are characterized by childhood neglect and high harm avoidance, avoidant PD has childhood anxiety disorders as risk factor, whereas borderline PD is characterized by childhood abuse, high novelty seeking, childhood depression and conduct disorder (Joyce et al., 2003). Further examination of the relation with specific EMSs in the disconnection/rejection domain for avoidant PD and borderline PD could lead to a better understanding of the overlap and differentiation.

Some EMSs were related to most of the PD traits, including social isolation and emotional inhibition in the borderline, avoidant and obsessive–compulsive PD dimensions. These findings indicate that isolation and inhibition might be considered a general and shared characteristic of PDs. PDs have indeed been found to have overlapping symptoms and traits, as well as high co‐morbidity(Trull & Durrett, 2005). This is also reflected by findings of the large overlap in relations when examining zero‐order correlations. The lack of relations between some of the PDs and EMSs in the regressions substantiate the shared variance among PDs. Other EMSs in our study however showed to be uniquely related to specific PD traits, for example, vulnerability to harm for borderline PD, emotional deprivation for avoidant PD, self‐sacrifice for dependent PD and unrelenting standards for obsessive–compulsive PD traits.

This suggests that PDs may be best reflected by a hybrid model of EMSs, with some EMSs that seem to reflect a broader vulnerability factor for PDs, whereas other EMSs are particularly relevant for specific PDs. As suggested by the current results, this hybrid model could incorporate overall beliefs of social isolation/alienation and emotional inhibition EMSs across PDs, which should be addressed globally in therapeutic interventions. Besides these, PDs are also differentially characterized by certain beliefs and domains, which could be specifically targeted in treatment for those PDs.

## 5 STRENGTHS AND LIMITATIONS

The current study has several strengths. First of all, PDs were represented dimensionally in this study, which is in line with the empirical evidence of an underlying dimensional, instead of categorical, nature of PDs (Arntz et al., 2009). Second, regression analyses between PDs and EMSs were examined using data from a rather large, clinical sample. Third, a structured clinical interview was used to diagnose clinical and PD disorders, instead of self‐reporting questionnaires as was done in other studies (Carr & Francis, 2010; Corral & Calvete, 2014; Meyer et al., 2001). Finally, in all analyses, several confounding variables were controlled for. These included both general characteristics like age, gender and education, as well as PD‐specificity by including all PD traits in the analyses. This allowed us to conduct more fine‐grained and specific analyses.

The current study also comes with several limitations. First of all, as we used an older version of the YSQ, we reported on 15, instead of 18, EMSs. Second, because of the non‐existent or very limited range of traits for some of the PDs (presence of *n* = 2 or less), six PDs had to be excluded from further analyses. It is not that surprising that paranoid, schizoid, schizotypal, narcissistic, antisocial and histrionic PDs were underrepresented in the current sample, as these PDs are generally less frequent in inpatient populations (Lenzenweger, Lane, Loranger, & Kessler, 2007). Furthermore, the current study did not examine additional variables that could influence the relation between EMSs and PDs, like metacognitive impairment (Semerari et al., 2014), reflective functioning and attachment styles (Levy et al., 2006). Finally, our sample consists of a population of highly educated clients, which may have influenced the number and type of PDs identified as socio‐economic status has been found to relate to mental health outcomes (Miech, Caspi, Moffitt, Wright, & Silva, 1999), as well as the present EMSs in this population. Future studies could include other factors that may help explain the association between EMSs and PDs, include all 18 instead of 15 EMSs as measured by the YSQ and examine relations with the current personality trait domains as captured by newer dimensional models (cf. ICD‐11 or DSM‐5 AMPD; Bach et al., 2018).

Taken together, the current study found borderline, dependent, avoidant and obsessive–compulsive PD traits to positively relate to a wide range of EMSs. This finding supports a more dimensional instead of categorical conceptualization of PDs, as the content of some EMSs seem to underpin more general PD pathology. One may question the relevance and necessity of the large number of differentiated PDs as identified by the DSM when thinking patterns as reflected by EMSs seem to largely overlap. There were however also several distinctive patterns, with vulnerability to harm for borderline PD, emotional deprivation for avoidant PD, self‐sacrifice for dependent PD and unrelenting standards for obsessive–compulsive PD traits. Therefore, the overall study findings support a hybrid model of PDs and EMSs. One clinical implication is that treatment targeting various schemas per PD is suitable. Furthermore, the same EMSs can be effectively related across various PD dimensions, as PDs do not seem to be differentially reflected by one single EMS only.

## CONFLICT OF INTEREST

There is no conflict of interest to be reported.

## Supporting information

Table S1. Description of Young's (1999) Early Maladaptive Schemas
