# Supplementary material for: Early maladaptive schemas and their relation to personality disorders: A correlational examination in a clinical population
Source: Clin Psychol Psychother. 2020 Jun 17;27(6):837–46. doi: 10.1002/cpp.2467 (PMC7754466; doi:10.1002/cpp.2467)
Supplement: Supplementary file 1 — Table S1. Description of Young's (1999) Early Maladaptive Schemas [file CPP-27-837-s001.docx]

| Table 1 | | |
| --- | --- | --- |
| *Description of Young’s (1999) Early Maladaptive Schemas* | | |
| Domain | EMSs | Description^1, 2^ |
| Disconnection/Rejection | Abandonment/Instability | Others will inevitably abandon me |
|  | Mistrust/Abuse | Others will abuse, hurt, lie, manipulate or take advantage of me |
|  | Emotional Deprivation | Others will not meet my need for emotional nurturance, empathy, and protection |
|  | Defectiveness | I am defective, bad, inferior or invalid |
|  | Social Isolation | I am different, alienated, an outsider |
| Impaired autonomy/Performance | Dependence | I cannot care for myself, need help from others |
|  | Vulnerability to Harm | Catastrophe will strike me, I will inevitably be harmed/injured/ill |
|  | Enmeshment | I am excessively involved with others (at the expense of full individuation) |
|  | Failure | I failed or will fail |
| Impaired Limits | Entitlement | I am superior to others, entitled to special rights |
|  | Insufficient Self-Control | I am unable to exercise self-control, have difficulty with delayed gratification |
| Other-Directedness | Subjugation | I am controlled by others because I feel coerced/avoid anger, abandonment and suppress my emotions/needs |
|  | Self-Sacrifice | I excessively focus on others’ needs at the expense of my own |
|  | Approval-Seeking | I excessively focus on gaining others’ approval/recognition |
| Overvigilance/Inhibition | Negativism | I focus on the negative aspects of life |
|  | Emotional Inhibition | I need to inhibit my emotions/feelings |
|  | Unrelenting Standards | I need to strive to achieve extremely high standards of behavior or performance |
|  | Punitiveness | People should be punished for mistakes |
| *Note.* ^1^Bernstein, Arntz, & Vos, 2007; ^2^Renner, Lobbestael, Peeters, Arntz, & Huibers, 2012. | | |
